# *EGFR* Mutations in Head and Neck Squamous Cell Carcinoma

**DOI:** 10.3390/ijms23073818

**Published:** 2022-03-30

**Authors:** Sindhu Nair, James A. Bonner, Markus Bredel

**Affiliations:** O’Neal Comprehensive Cancer Center, Department of Radiation Oncology, The University of Alabama at Birmingham Heersink School of Medicine, Birmingham, AL 35233, USA; nairsin8@uab.edu

**Keywords:** EGFR, head and neck squamous cell carcinoma, kinase inhibitors, resistance

## Abstract

EGFR is a prototypical receptor tyrosine kinase that is overexpressed in multiple cancers including head and neck squamous cell carcinoma (HNSCC). The standard of care for HNSCC remains largely unchanged despite decades of research. While EGFR blockade is an attractive target in HNSCC patients and anti-EGFR strategies including monoclonal antibodies and kinase inhibitors have shown some clinical benefit, efficacy is often due to the eventual development of resistance. In this review, we discuss how the acquisition of mutations in various domains of the EGFR gene not only alter drug binding dynamics giving rise to resistance, but also how mutations can impact radiation response and overall survival in HNSCC patients. A better understanding of the EGFR mutational landscape and its dynamic effects on treatment resistance hold the potential to better stratify patients for targeted therapies in order to maximize therapeutic benefits.

## 1. Introduction

The epidermal growth factor receptor (EGFR) is a member of the ErbB/HER family of receptor tyrosine kinases (RTKs) and is essential for several cellular survival processes [1,2]. An extensively studied biomarker, *EGFR* is one of the most implicated genes in carcinogenesis due to its frequent overexpression and mutations in multiple cancers [3,4]. Head and neck squamous cell carcinomas (HNSCC) are tumors that arise predominantly in the mucosa of the oral cavity, sinuses, oropharynx, hypopharynx, and larynx [5]. Up to 80–90% of HNSCCs overexpress or harbor mutations in EGFR, and these alterations directly impact overall and progression-free survival [6,7,8]. Targeting EGFR therapeutically using anti-EGFR monoclonal antibodies or kinase domain inhibitors with concomitant radiation remains one therapeutic option for patients with HNSCC [7,9,10]. However, the efficacy of these therapies can be compromised, on the one hand, due to the presence of pre-existing genetic alterations in *EGFR* that render them resistant to EGFR blockade or, on the other hand, the acquisition of secondary mutations under therapeutic pressure, which helps evade targeting [11,12]. EGFR status is increasingly being recognized as a predictor of survival as well as chemoradiation response in HNSCC. In this review, we provide an overview of various genetic alterations in *EGFR* and how these mutations impact chemoradiation response as well as survival in HNSCC.

## 2. The EGFR Structure

**Figure 1:**
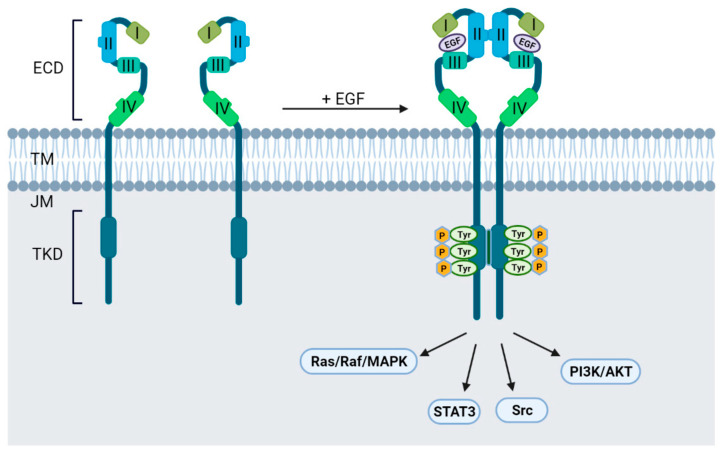
EGFR structure with relevant domains and signaling activation.

EGFR is a transmembrane protein comprised of an extracellular ligand-binding domain (ECD), a transmembrane domain (TD), a juxtamembrane (JM) segment, a tyrosine kinase domain (TKD), and a C-terminal regulatory tail [13,14]. The structure of EGFR with relevant domains is depicted in Figure 1. The ECD of EGFR is composed of four domains necessary for ligand binding. In an inactive state, domains I, II and III adopt a ‘closed’ conformation, while in an active state, EGF binds to the pocket between domains I and III and favors a conformational change to an ‘open’ untethered state [15]. This rearrangement allows domains II and IV to bind to the corresponding domains on the adjacent receptor leading to homo- or hetero-dimerization. The conformational change caused by ligand binding leads to auto- and trans-phosphorylation of the TKD with subsequent recruitment of adaptor proteins such as Src homology 2 (SH2) or phosphotyrosine-binding (PTB) domains. These domains then activate downstream pathways essential for cell survival and proliferation [15,16,17,18]. Since EGFR plays a critical role in regulating various cellular processes, oncogenic mutations in any of these domains result in aberrant expression and/or dysregulated signaling while also altering responses to EGFR targeting agents.

## 3. EGFR Mutations and Drug Resistance

**Figure 2:**
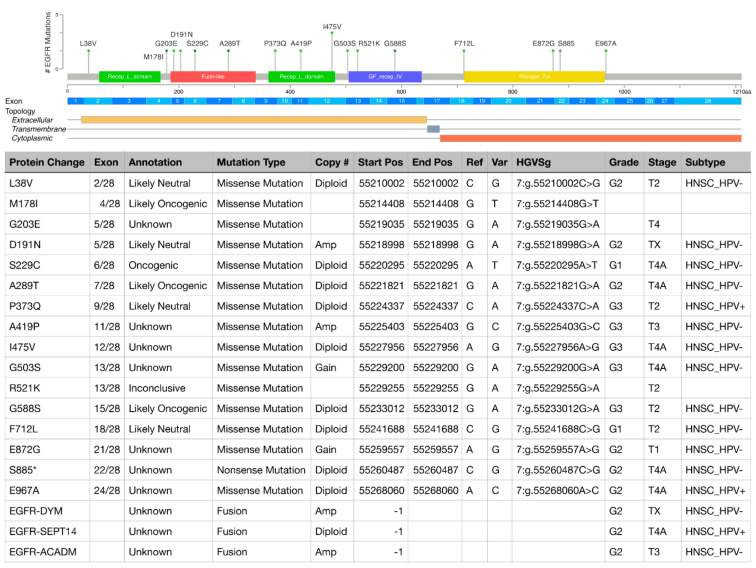
*EGFR* mutations and gene fusions in HNSCC patients curated in the cBioPortal for Cancer Genomics in relation to *EGFR* exons and EGFR protein domains. The table shows details of the specific mutations and corresponding patients, including protein change, exon location, functional annotation according to OncoKB precision oncology knowledge base, type of mutation, *EGFR* gene copy number, mutation site, nucleotide change (reference [Ref] vs. variant [V]), Human Genome Variation Society genomic nomenclature (HGVSg), tumor grade, tumor, stage, HPV subtype (+, positive; −, negative). *EGFR* mutational and clinical data were visualized, analyzed, and downloaded from the cBioPortal for Cancer Genomics (https://www.cbioportal.org; accessed on 19 March 2022 [22,23]).

Acquisition of treatment resistance can be due to diverse mechanisms including (a) pre-existing mutations in *EGFR* or those acquired during therapy, (b) aberrant expression of EGFR ligands, (c) defective endocytosis of EGFR, (d) alterations in downstream signaling cascades, (e) receptor switching or crosstalk with other RTKs, and (f) epigenetic modifications [19,20,21]. Additionally, tumors develop resistance by acquiring mutations in domains where therapeutic antibodies bind. Multiple mutations have been reported in the TKD and the ECD, regions that are targeted for EGFR blockade (Figure 2); mutations in the TM domains have not been reported in HNSCC so far. Here, we discuss how mutations in *EGFR* domains initiate and drive resistance in HNSCC.

### 3.1. Extracellular Domain Mutations

Ectodomain targeting monoclonal antibodies against EGFR competitively bind and terminate signaling by binding to domain III of the ECD. Commonly used monoclonal antibodies include the FDA approved Cetuximab (CTX) and Panitumumab (PAN) with the former exhibiting higher efficacy in combination with radiotherapy or other biologic and chemotherapeutic agents such as pembrolizumab or cisplatin [9,24,25]. Until now, mutations reported in the ECD of *EGFR* have exclusively been associated with CTX resistance in HNSCC while mutational resistance to PAN has been less explored with scant reports [20,26].

**Figure 3:**
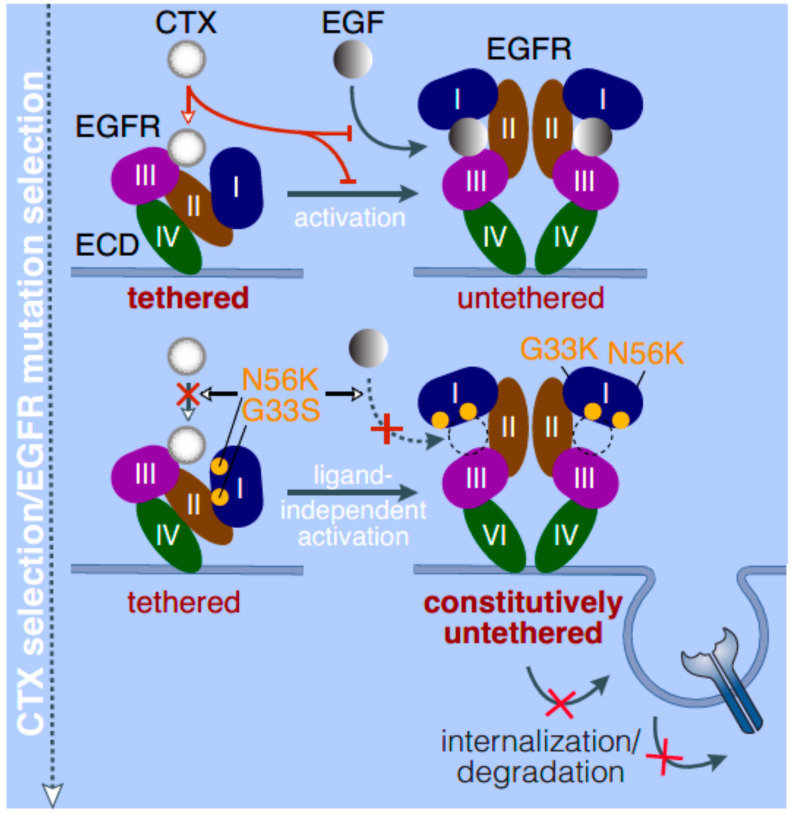
CTX and EGF binding dynamics in normal cells and cells with ECD mutations in EGFR.

A clinical study investigating the development of resistance in a patient initially sensitive to CTX analyzed both pre- and post-CTX tumor biopsies and found a missense mutation in the EGFR-ECD at the 465th position with a glycine to arginine substitution (G465R) [26]. Further analysis found that the mutation altered the binding of CTX to domain III of the ECD sustaining resistance. Additional non-synonymous mutations (alterations in amino acid sequence) have been reported in the ECD of EGFR (G33S, N56K) in an HNSCC cell line selected for resistance to CTX (20). These acquired mutations in domain I of the ECD resulted in a lower affinity for EGF binding; however, constitutive activation of EGFR was observed along with diminished internalization of EGFR for degradation. Additionally, these mutations trapped EGFR in an open extended conformation that prevented CTX from accessing its binding site on domain III leading to resistance [20] (Figure 3). Interestingly, a similar mechanism has been observed with *EGFR* ECD mutations in glioblastoma, where missense mutations located at the domain I-to-II interface led to spontaneous untethering of the self-inhibitory tether driving oncogenicity [27]. Additionally, the *A289T* mutation (Figure 2) has been reported in glioblastoma multiforme, anaplastic astrocytoma, and lung adenocarcinoma [28] and appears to favor a ligand-independent formation of the active state [29]. Therefore, ECD mutations in *EGFR* across tumors seem to share a common mechanism of bypassing steric inhibition leading to unchecked signaling as well as the acquisition of a resistant phenotype by preventing monoclonal antibody binding to EGFR. CTX resistance has also been reported in patients with an EGFR-K_521_ (K-allele) polymorphism, in which CTX had lower binding affinity along with an inability to inhibit downstream signaling [30]. The authors suggested this polymorphism could be used as a prognostic predictor of therapeutic resistance, which is discussed in subsequent sections of this review. While most mutations are acquired in response to therapy, one report showed that the presence of a R521K substitution in both in vitro and in vivo HNSCC models rendered tumors resistant to CTX but sensitive to a c-MET TKI (SU11274) [31]. Interestingly, a discrepancy in EGFR expression between the primary tumor and metastatic brain lesions was observed in a patient under long-term CTX treatment who eventually acquired resistance [32]. The metastatic lesions had lower *EGFR* expression and higher expression of N-cadherin with an upregulation of epithelial-to-mesenchymal (EMT) transition genes indicating a mechanism of therapeutic evasion. In general, overcoming resistance to CTX entails switching to a different monoclonal antibody or targeting alternative domains in EGFR such as the tyrosine kinase domain as discussed below.

### 3.2. Tyrosine Kinase Domain Mutations

Structurally, the TKD of EGFR is a vital component and mediator of downstream signaling cascades that regulate diverse biological processes [33]. Targeting the TKD is an attractive approach as cancer cells are dependent on RTK signaling for sustenance. TKIs bind to the TKD and inhibit phosphorylation of the kinases preventing downstream signal transduction [19]. Several kinase targeting therapies called tyrosine kinase inhibitors (TKIs) such as Gefitinib, Erlotinib, and Afatinib, have been used to treat HNSCC in the past few decades [9,34,35,36]. As with ECD targeting therapies, the eventual development of resistance to TKIs is a major factor in limiting their efficacy; aberrant activation of the TKD mediated by mutations is a mechanism exploited by cancers to not only propagate signaling but also evade TKI targeting.

A retrospective study of 47 diagnosed HNSCC cases in a Saudi cohort showed that 57% of tumors had mutations in the TKD spanning exons 18 to 21. Specifically, a T790M mutation was observed in 4 patients resistant to tyrosine kinase inhibitor (TKI) therapy. Mutational status also correlated with higher grade and advanced stage of the tumor [37]. Conversely, mutational screening of 52 specimens in a Belgian cohort did not find any missense mutations in the TKD indicating that the prevalence of TKD mutations could possibly vary between different ethnic populations [38]. A systematic review of 53 studies found the overall pooled prevalence of TKD mutations in head and neck cancer patients was 2.8% in 4122 patients studied. It was discovered that 41.5% of these mutations were observed in exon 19, 32.1% in exon 20, 17% in exon 21, and 9.4% in exon 18. The predominant mutation was of the missense type with T790M and L861Q substitutions observed in multiple cases [39]. The T790M mutation has also been reported as the most prevalent resistance alteration in TKI resistant lung carcinoma patients suggesting that the mutational profile with respect to TKI resistance could be shared across tumors [12,40,41]. Kinase domain duplication (KDD), which entails duplication of the exons encoding the TKD, has been reported in HNSCC [42]. These KDD mutants exhibit higher phosphorylation as well as EGF- independent activation states leading to aberrant EGFR signaling [42]. Overall, somatic *EGFR* mutations and gene copy gain mediate therapeutic resistance in HNSCC underscoring the need for newer strategies such as multitargeted kinase inhibitors or dual targeting of both the ECD and the TKD [36,43,44,45].

## 4. EGFR Alterations and Radiation Response

For many HNSCCs, the response to a single-agent chemotherapy or monoclonal antibody alone remains dismally low mandating a multi-modal approach combining surgery, chemotherapy and/or radiation [46,47]. Early in vitro studies demonstrated that high concentrations of EGF slowed tumor growth, presumably through negative feedback and resulted in radiosensitization of human HNSCC cells [48,49]. Next, anti-EGFR blocking antibodies, which inhibited EGFR-induced signaling, showed prominent radiosensitization for squamous cell carcinomas in vitro [50,51,52]. Subsequently, patients with locoregionally advanced HNSCC treated with concomitant radiotherapy (RT) plus CTX exhibited a significantly improved 5-year survival and locoregional control without major toxic effects [24,53]. Multiple studies examining the role of EGFR in radiation resistance of HNSCC found that patients with high expression of EGFR had significantly lower overall survival and higher relapse rates [54,55]. At a cellular level, EGFR also modulates the repair of radiation-induced double stranded DNA breaks (DSB) by forming an EGFR–DNA-PK complex [56]. Inhibition of EGFR by CTX in HNSCC increased radiation-induced apoptosis and dysregulation of repair mechanisms mediated by downstream effectors such as JAK-STAT3 and PI3K-AKT pathways confirming the role of EGFR in mediating radioresistance [57]. One study reported that prolonged treatment with CTX promoted p27^Kip1^-mediated G1 arrest with the induction of autophagy [58]. The activation of autophagy has been shown to have opposing roles with respect to radiation response with some reports showing autophagy to be a radiosensitizer [59,60], while others show it plays a role in radioresistance [61]. Interestingly, a recent study found that EGFR expression and RT response depended on the human papilloma virus (HPV) status of the tumors [62]. In HPV-negative HNSCC cells, EGFR overexpression conferred increased survival, epithelial-to-mesenchymal transition and radioresistance via activation of vital DSB repair proteins post-irradiation. Conversely, in HPV-positive HNSCC cells, EGFR overexpression increased radiosensitization by abrogating the expression of DSB repair proteins post-irradiation. Additionally, HPV E16 levels were significantly suppressed in EGFR overexpressing HPV-positive cells resulting in restoration of p53 activity leading to RT sensitivity. When EGFR was inhibited using the TKI Gefitinib, it led to radiosensitization, further confirming that EGFR expression has a vital role in RT response [62].

EGFR also plays indirect roles in radioresistance by exploiting various oncogenic survival mechanisms. For example, EGFR is known to regulate and maintain cancer stem cells (CSC), which are distinct subpopulations in tumors with the ability to self-renew and differentiate, and which are known to be inherently radioresistant due to their enhanced DNA damage repair abilities [63,64,65]. As a result, discrepancies in RT sensitivity exist within a tumor leading to radioresistance and relapse. Two separate studies found that combining the EGFR inhibitor afatinib with ionizing radiation led to a significant decrease in CSC populations as well as colony forming abilities of these CSCs with an overall decrease in tumor size [66,67]. An increase in phosphorylated γH2AX, indicative of DNA damage, was also observed in afatinib treated cells [66,67]. Another study explored the role of HIF-1α, a marker of hypoxia, which is known to upregulate EGFR expression in tumors [68]. When HIF-1α was inhibited using rapamycin and used in combination with CTX and concomitant ionizing radiation in HNSCC in vivo models, a significant reduction in tumor sizes were observed initially followed by a rapid relapse. The authors found that this treatment regimen induced the expression of another hypoxia gene known as HIF-2α, which mediates resistance to EGFR inhibition as well as radioresistance. Subsequent HIF-2α inhibition with concomitant EGFR inhibition and ionizing radiation led to radiosensitization and a significant decrease in tumor growth [68].

Taken together, EGFR mediates radiation response in HNSCC either directly, by regulating its own downstream activation partners or DNA damage response proteins, or indirectly, by initiating and regulating tumor-specific survival mechanisms.

## 5. EGFR Alterations as Prognostic Indicators for Disease and Therapeutic Response

**Figure 4:**
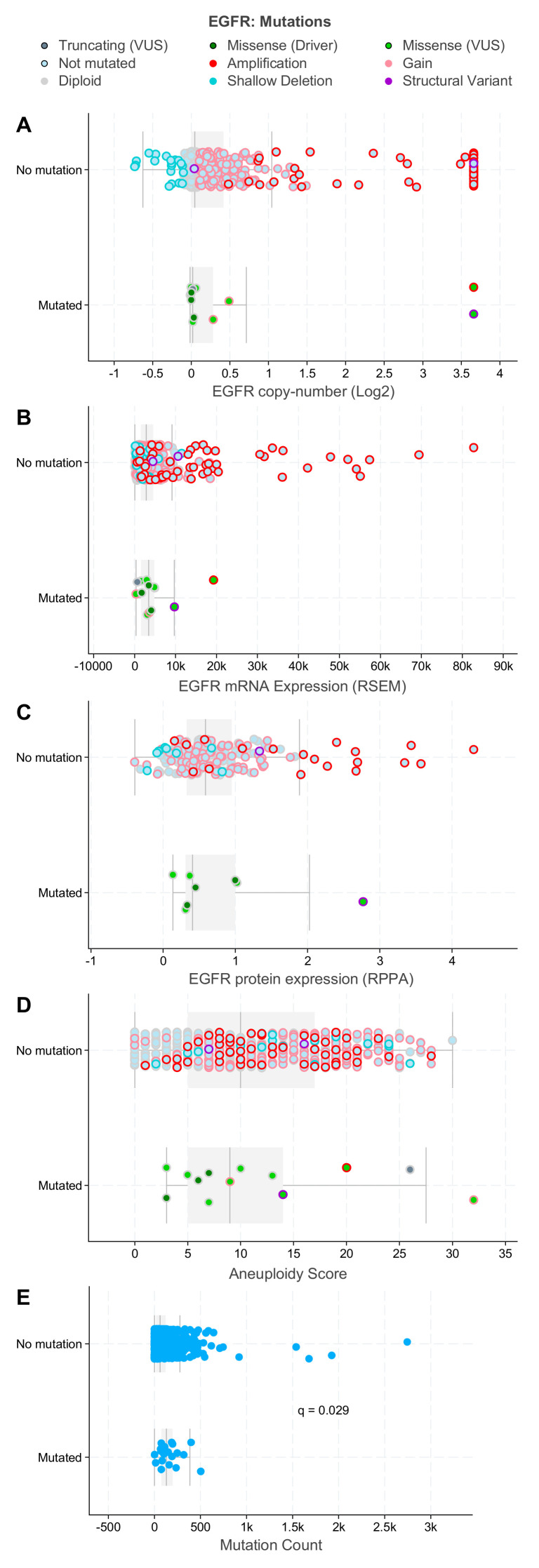
*EGFR* mutations in relation to *EGFR* copy number (**A**), mRNA (**B**), protein (**C**), and genome-wide aneuploidy (**D**) levels and overall mutational burden (**E**) in HNSCC patients curated in the cBioPortal for Cancer Genomics. *EGFR* mutations rarely co-segregate with *EGFR* amplifications (**A**) and show ranges of EGFR mRNA (**B**) and protein (**C**) expression comparable to their non-amplified counterparts. *EGFR* mutated tumors show a range of chromosomal aneuploidy scores (**D**) similar to non-mutated tumors, but demonstrate a significantly lower overall mutational burden (**E**). *EGFR* genomic, and aneuploidy, and mutational count data were visualized, analyzed, and downloaded from the cBioPortal for Cancer Genomics (https://www.cbioportal.org; accessed on 19 March 2022 [22,23]).

**Figure 5:**
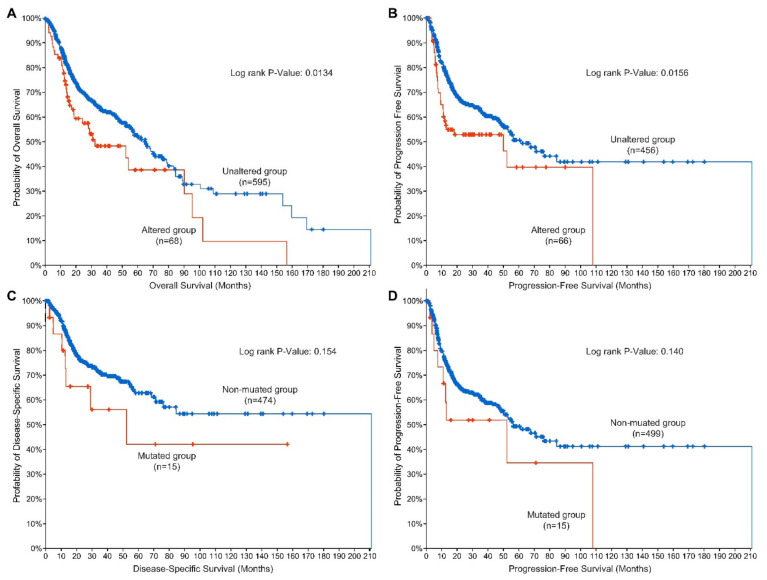
*EGFR* alterations and survival in H&N cancer patients curated in the cBioPortal for Cancer Genomics. Patients who carry *EGFR* mutations and/or amplifications demonstrate a significantly briefer overall (**A**) and progression-free survival (**B**) than patients with unaltered *EGFR*. *EGFR* mutations are suggestively associated with disease-specific (**C**) and progression-free survival (**D**). *EGFR* genomic and clinical data were visualized, analyzed, and downloaded from the cBioPortal for Cancer Genomics (https://www.cbioportal.org; accessed on 19 March 2022 [22,23]).

With the importance of EGFR in mediating chemoradiation response discussed in the previous sections, EGFR has been shown to be of prognostic significance in HNSCC. The predictive value of EGFR has been very well established using various methodologies such as immunohistochemical analysis of tumor samples, fluorescent in situ hybridization, tissue microarray, and gene sequencing [69,70,71]. Multiple studies have shown that the overexpression of EGFR in HNSCC directly correlates with worse outcomes [47,54,72,73,74]. Meta-analyses have shown that EGFR overexpression is associated with reduced overall survival (OS), and progression-free survival (PFS), and disease-free survival (DFS) with the magnitude of these effects varying from study to study [75,76,77,78]. *EGFR* mutations appear to rarely co-segregate with amplifications of the *EGFR* gene (Figure 4) in H&N cancers as is often the case in glioblastoma. *EGFR* mutated HNSCCs show a ranges of EGFR mRNA and protein expression comparable to their non-amplified counterparts. *EGFR* mutated tumors show a range of chromosomal aneuploidy scores similar to non-mutated tumors but demonstrate a significantly lower overall mutational burden (Figure 4). Patients with either *EGFR* mutation or amplification demonstrate comparatively briefer overall survival and progression-free survival than patients with tumors that do not carry these genetic events, indicating that these alterations could have potential prognostic value. Moreover, *EGFR* mutations are suggestively associated with disease-specific and progression-free survival in H&N cancers (Figure 5). While these observations are based on univariate analyses and thus have obvious limitations, they are hypothesis-generating for future work. The low variant frequency for the *EGFR* mutation—a situation in which variance estimates tend to inflate—might make it difficult to model this survival relationship in an adjusted model unless a very large sample size is studied.

A phase III EXTREME study that evaluated *EGFR* copy number as a predictive marker in patients treated with a combination of CTX and platinum/5-fluorouracil (5-FU) found no association between copy number and OS or PFS [79]. Therefore, in certain situations, EGFR expression may be helpful in discussing outcomes for patients, but this line of investigation needs further discernment.

From a mutational perspective, EGFR status has had diverse effects based on the treatment regimens used. Two separate studies examined the presence of a single nucleotide polymorphism (SNP) EGFR-K_521_ in different HNSCC clinical cohorts and found that while the SNP was not associated with the risk of cancer, it correlated with response to CTX treatment [30,80]. In vitro studies showed that HNSCC cells with a R521K substitution in the ECD did not respond to CTX treatment; cell proliferation and apoptosis remained unimpacted in both in vitro and in vivo models indicative of intrinsic resistance to CTX [31].

Several *EGFR* gene polymorphisms have been associated with an elevated HNSCC risk. Genotyping of 578 HNSCC patients and 588 cancer-free controls for 60 *EGFR* SNPs revealed intronic SNPs rs12535536, rs2075110, rs1253871, rs845561, and rs6970262, and synonymous SNP rs2072454 were associated with HNSCC risk among all HNSCC patients [81]. The EGFR-R497K substitution has been reported to be an independent predictor for both OS and therapeutic response; patients with this SNP exhibited lower response to CTX treatment [82] but had a decreased risk of disease-specificity mortality [83]. On the contrary, an in vitro study found that HNSCCs carrying the EGFR-R497K mutation were more likely to be susceptible to CTX treatment [84]. These discrepant findings may be attributed to a differing experimental approach, namely, an in vitro versus an in vivo setting.

The *EGFR* mutational spectrum in non-small cell lung cancers (NSCLC) is very well defined with respect to therapeutic response. Systematic reviews in NSCLC show how patients with certain mutations exhibit differential sensitivities and clinical outcomes to TKIs and immunotherapy [85,86,87,88]. In general, most mutations involving exons 18, 19 and 21 are predictive of sensitivity to TKIs while exon 20 mutations are considered predictors of resistance in NSCLC [89,90,91,92,93]. For example, several case studies have shown that exon 19 deletions and exon 21 substitutions such as L858R, which account for approximately 50% of EGFR mutations in NSCLC, are known sensitizers to first and second generation TKIs [94,95,96,97]. Exon 20 substitutions such as T790M and C797S, most implicated in resistance to gefitinib and erlotinib, have been shown to be susceptible to third generation TKIs such as alflutinib and osimertinib with varied efficacies in different patient cohorts [98,99,100,101,102]. Since mutational patterns could be shared between tumors, some studies have attempted to screen for these specific mutations in HNSCC cohorts in order to assess chemoradiation response. A systematic review of a 113-patient HNSCC cohort found that the L858R substitution, associated with sensitivity to EGFR TKIs in NSCLC, was found in only 2.5% of the patients; the T790M substitution in exon 20, was found in 7.5% of patients; and in-frame deletions in exon 19, making up 45% of all *EGFR* mutations in NSCLC and linked to responsiveness to EGFR TKIs, were observed in 22% of all *EGFR*-mutated HNSCCs (37). This indicates that some of the predictive value of *EGFR* mutations, which are known predictors of therapeutic response in NSCLC, could be extrapolated to HNSCC. However, large-scale screening in multiple treatment cohorts as well as detailed analysis is required to demonstrate the prognostic capabilities of *EGFR* mutations in HNSCC.

Taken together, *EGFR* genotyping could prove to be useful in predicting response to EGFR-targeted therapies in HNSCC and help stratify patients to escalated or deescalated treatment regimens.

## 6. Conclusions

Despite the latest advancements in EGFR-targeted therapies, the presence or absence of *EGFR* mutations plays a role in the response of tumors to various treatments. More research is needed to better define the mutational landscape of EGFR and its dynamic biologic effects in HNSCC as a means to tailor EGFR-targeted therapies and thus enhance the clinical benefit derived from these therapies.

## Data Availability

Not applicable.
